# Cysts in the Floor of the Mouth

**Published:** 1908-01-25

**Authors:** 


### CYSTS IN THE FLOOR OF THE MOUTH.

The term ranula is now used exclusively in reference to the bluish-grey cystic swellings found under the mucous membrane of the floor of the mouth which are supposed to be due to obstruction of one or other of the salivary ducts. Mr. Bland-Sutton suggests that they should be called respectively sublingual, submaxillary, and parotid ranulæ according to the duct affected, and he believes that some pathological factor is responsible for their production apart from mere mere obstruction. It has been supposed that some of them at any rate are due to cystic degeneration of the glands of Blandin and Nuhn. This certainly is the most reasonable explanation of some of the smaller mucous cysts found in this situation. Others suppose that the cause is to be found in subacute inflammation of the bursa situated between the mucous membrane and the genio-hyo-glossus muscle. The cyst is lined by flattened epithelial cells, and contains a thin mucoid fluid which consists of altered saliva.

The best treatment is to make an incision in the mucous membrane over the cyst, and to dissect it out. It can rarely be removed in its entirety, for the cyst wall is so thin that it usually gives way when it is manipulated, and discharges its contents. The whole cyst wall should, if possible, be taken away. It is not necessary to put any stitches in the mucous membrane. If the entire cyst wall cannot be removed, as sometimes happens, the cavity must be lightly plugged, and allowed to heal by granulation. It is not sufficient to puncture the cyst and let out its contents, as used to be taught: if this is all that is done, the wall secretes more fluid and the condition recurs. The name of acute ranula is sometimes applied to a painful swelling of the submaxillary gland itself, due to obstruction of Wharton's duct by a calculus. The swelling varies in size from time to time, and enlarges with the desire for food. If the condition is left, the calculus may become as large as a cherry. It is usually composed mainly of calcium phosphate. Sooner or later the gland becomes inflamed by reason of infection from the mouth, and it is this which makes the swelling painful. The treatment is quite simple: it is to cut down on the stone and remove it. The condition does not tend to recur.

Cysts are found in connection with the thyroglossal duct. This structure is, in the embryo, the duct of the thyroid gland; but it atrophies as development proceeds, and its only remnant in a normal individual is the foramen cæcum on the dorsum of the tongue. The line of the duct in the embryo is from the dorsum of the tongue, between the geniohyo-glossi, behind the body of the hyoid bone to the isthmus of the thyroid gland. Two forms of cyst are described in connection with it; a simple retention cyst of the persistent remains of the lingual part of the duct. This is lined with cubical epithelium and contains a colloid brown material. It is generally situated deep in the muscles of the tongue, and may easily be mistaken for a gland. It should be removed by operation. If situated deeply, it is better to attack it through a median submental incision dividing the mylo-hyoid muscle. It is important to remove it completely. If this precaution is not observed, a troublesome fistula is often the result. Dermoid cysts originate also in the thyro-glossal duct. These are more superficial, and arise in connection with the extreme lingual end of the duct. The nature of these cysts is not always diagnosed before they burst or are removed by operation. But their position at the site of the foramen cæcum should cause one to suspect their nature. Like dermoid cysts generally, they are lined by squamous epithelium, and contain broken-down sebaceous material, cholesterin crystals, and sometimes a few hairs. The treatment is simple: they should be removed by operation.

